# A Cross-Sectional Study to Examine Factors Associated with Primary Health Care Service Utilization among Older Adults in the Irbid Governorate of Jordan

**DOI:** 10.1155/2014/735235

**Published:** 2014-11-06

**Authors:** Abdullah Alkhawaldeh, Margo B. Holm, Jamal Qaddumi, Wasileh Petro, Madi Jaghbir, Omar Al Omari

**Affiliations:** ^1^Faculty of Nursing and Midwifery, Jerash University, Amman, Irbid International Street, Jerash 26150, Jordan; ^2^Faculty of Health and Rehabilitation Sciences, University of Pittsburgh, Pittsburgh, PA 15260, USA; ^3^Faculty of Medicine and Health Sciences, An-Najah National University, P.O. Box 7, Nablus, Palestine; ^4^Faculty of Nursing, Jordan University, Amman 11942, Jordan; ^5^Faculty of Medicine, Jordan University, Amman 11942, Jordan

## Abstract

*Background*. Recently, the percentage of older adults in developing countries has increased significantly. *Objective*. This study examined patterns and factors associated with primary health care services utilization in the past 1, 6, and 12 months. *Method*. A cross-sectional study design was used to collect data from 190 older adults in the Irbid governorate of Jordan. *Results*. Primary health care services were used by less than half of the participants in the past 1 month, by 68.4% in the past 6 months, and by 73.8% in the past 12 months. Primary health care (PHC) services use was associated with age, education level, tobacco use, chronic illnesses, perceived general health status today, a physical component summary score, employment, and perceived general health status in the past 6 and 12 months. The primary predictor of PHC services use at 1, 6, and 12 months was chronic illnesses (OR = 13.32), (OR = 19.63), and (OR = 17.91), respectively. *Conclusion*. Although many factors were associated with PHC service utilization, the strongest predictor of PHC service utilization was chronic illnesses.

## 1. Introduction

Recently, the percentage of older adults in developing countries has increased significantly [1]. Middle income countries have found that this increase in older adults represents a major challenge for health care organizations as a result of the physical, social, and psychological changes associated with complex morbidity and mortality profiles [2]. Subsequently, all countries need to be prepared to address the consequences of the demographic trends associated with aging populations. Worldwide, the increasing health care demand of older persons requires a better understanding of factors influencing health care service utilization patterns [3]. In several parts of the developing world, primary health care (PHC) use is inadequately understood [4]. In Jordan, there is an inadequate amount of reliable data existing on health care utilization rates and thus health care planners are unable to estimate accurately the real needs of the population [5].

Some studies have explored health services utilization patterns across several countries [6–8]. A study of older Estonians found that about 81 percent indicated having visited a general practitioner or specialist during the prior 1 year [9]. Similarly, health service utilization among older Nigerians was high, finding that 95.3% had visited a health care facility and 90.3% reported being sick frequently; however, just 67.8 percent visited a health facility once they were critically sick [6]. In contrast, health service use among older Chinese in the past 6 months was less than 50 percent and consisted of general outpatient clinic services (36.7%) and specialist outpatient clinic services (20.8%) [7]. Similarly, health care services use in the past 1 month among older adults in Hong Kong was less than 50 percent and the services were provided by Western medicine physicians (both generalists and specialists) in a Government clinic (31.1%), private physicians of Western medicine (11.1%), practitioners of Chinese medicine (2.5%), and Western medicine practitioners in emergency units (1.9%) [8]. In Spain, researchers found that, of the health care services utilized by 74.5% of older adults in the past 3 months, 18.4% were nursing staff visits, 59.4% were general practitioner visits, and 16.5% were specialist visits [10].

Patterns in health services utilization by older adults have been found to be associated with many factors [7–13]. For example, among 787 older adults over 64 years of age in Spain, health service contacts were greater for females than males. Moreover, educational level was negatively associated with outpatient services, chronic diseases, and medication use. Additionally, poorer health status was positively associated with general practitioner consultations, smoking was negatively associated with health care use, and older adults with depression or the perceived need for care were associated with greater health care use. In Spain, the significant predictors of general practitioner use were perceived need for care, self-reported health status, and educational level [10]. In Hong Kong, a study found that age was significantly associated with hospital use, income was significantly associated with both general and specialist outpatient clinic services utilization, and family instrumental care was significantly associated with hospital use. Self-rated health status and particular kinds of chronic illness were significantly associated with use of the four services. Also, poorer activities of daily living scores were associated with both emergency room and general outpatient clinic use, and greater symptoms of depression were associated with hospital use [7].

A study conducted in Ghana reported that health care utilization was associated with participants' age group, occupation, medical history of chronic conditions, cognitive impairment in past 30 days, and self-reported health status. In addition, significant predictors were age group, medical history of chronic condition, cognitive impairment, self-perceived health, and difficulty with picking up items in the past 30 days [13]. In Korea, a study found that those most likely to have used long-term care services were 80 years and older, had less than 13 years of education, lived with others, were religious, had no income and health insurance, had poor health status, and had activities of daily living/instrumental activities of daily living (ADL/IADL) limitations and cognitive impairment. Determinants of long-term care services use were health status, ADL/IADL limitations, and cognitive impairments [12]. A Thai study found that greater health care services utilization in the past year, among 504 adults aged 60 and older, was associated with older males [11]. The utilization of health care services was also connected with married, less educated participants, and diagnoses with a chronic illness [11].

Although there were some studies that studied health status of older adults in Jordan [14–19], a comprehensive literature review revealed a dearth of research regarding factors that influence PHC service use among older adults in Jordan. Hence, this study aimed to examine patterns and factors associated with PHC utilization by Jordanians aged 50 years and older living in the Irbid governorate of north Jordan.

## 2. Materials and Methods

### 2.1. Design

A cross-sectional study design was used to examine PHC services utilization patterns and to identify factors associated with and predictive of health care utilization/nonutilization in the past 1, 6, and 12 months. The study was conducted in the catchment areas associated with three comprehensive PHC centers which provide services from 8 a.m. to 4 p.m., and most of these health centers serve large numbers of people and offer many preventive and curative health care services situated in areas within the Irbid governorate of north Jordan [20].

### 2.2. Participants

Adequate sample size needed for binary logistic regression [21] was determined using the formula proposed by Peduzzi et al. [22]. They suggested a minimum *N* that is at least 10 times *K*, where *K* is the number of predictors in the model. Using their criteria, the minimum sample size needed for this study was 190 participants.

A proportional convenience sample of 190 older adults, aged 50 and older, participated in this study from three areas (south (*n* = 79), center (*n* = 85), and north (*n* = 26)) of the Irbid governorate in Jordan (Irbid is the third largest inhibited city). These primary health care centers serve around 69,088 inhabitants.

### 2.3. Measures

#### 2.3.1. Dependent Variables

PHC service utilization in the past 1, 6, and 12 months.

Participants were asked the following. Did you visit the primary health care center in your region during the past month? Past 6 months? Past 12 months?

#### 2.3.2. Independent Variables

Based on Anderson's behavioral model, predisposing, enabling, and need factors were included as follows [23]: predisposing factors included age (years), gender (male or female), health behavior measuring tobacco use (smoker or nonsmoker), employment status (unemployed, retired, and employed), education level (no education, primary school, and secondary), and marital status (married, single, separated or divorced, and widowed). Enabling factors included monthly income and health insurance coverage (insured or not insured). Need factors included chronic illness self-reports (have/do not have a chronic illness).

Additionally, cognitive impairment was measured by using the Elderly Cognitive Assessment Questionnaire (ECAQ) [24]. Perceived general health was measured in two ways: (1) on a scale of 1 to 10, with a 1 representing the “worst I have ever felt” and a 10 representing the “best I have ever felt.” What number would best represent your general health today? 6 months ago? 12 months ago? And (2) perceived general health status in the past 1-month period was measured using the 12-Item Short Form Health Survey version 2 (SF-12v2) [25]. SF-12v2 measures eight health domains: physical function, role-physical, bodily pain, general health, vitality, social functioning, role-emotional, and mental health. These domains are summarized as physical component summary (PCS) and mental component summary (MCS) scales and use norm-based scoring. When the scores are transformed, the general population has a mean of 50 and a standard deviation of 10. So, when compared to the general population, health related quality of life (HRQOL) is considered to be lower than the norm if PCS or MCS scores are calculated to be lower than 50 [25].

### 2.4. Data Analysis

Descriptive statistics were used to describe study variables. Chi-square associations with categorical variables and Pearson correlations were conducted to establish associations between independent and dependent variables and to identify which variables would enter logistic regression model. Correlation tests included were Spearman's rho, Point Biserial *r*, Phi coefficient (*ϕ*), and Cramer's V and were used depending on the level of measurement for each variable. Three binary logistic regression models were developed for utilization/nonutilization of PHC services during the past 1, 6, and 12 months. The probability (*P* < 0.05) was taken as minimum level of significance.

### 2.5. Ethical Considerations

Permission for conducting study was obtained from the University Of Jordan School Of Nursing and the Jordan Ministry of Health Ethical Committee. Permission to use the ECQA instrument was obtained from the instrument developer and permission to use the Algerian (Arabic) SF-12v2 was obtained from Quality Metric Incorporated (License agreement CT130430/OP011094). All participants were notified that the data collected would be treated with anonymity and confidentiality. In addition, personal informed verbal consent was obtained from all participants.

## 3. Results

### 3.1. Participants

Table 1 depicts participants' characteristics. The mean age of participants was 64.6 years (SD = 9.7). There were more male (57.4%) participants, and the majority of participants were married (88.4%). About 36.8% of participants had no formal education, although 42.1 percent of the participants received a primary school education and 21.1 percent received a secondary education or higher. Most of the participants were unemployed (55.3%) and nonsmokers (71.1%). The majority (93.7%) had some type of health insurance coverage. The mean monthly income per participant was 218.2 Jordanian Dinars. About 72.1% of participants had chronic illnesses and 96.8% showed no evidence of cognitive impairment. For perceived general health status on the SF-12v2 for the past 1 month, the mean physical component summary (PCS) score was 41.28 (SD = 11.0) and 50.46 (SD = 7.3) for the mental component summary (MCS) score. For perceived general health status today, in the past 6 and 12 months, the means were 6.61 (SD = 1.3), 6.46 (SD = 1.3), and 6.64 (SD = 1.3), respectively.

### 3.2. Older Adult PHC Services Utilization Patterns

In the past 1, 6, and 12 months, 41 percent of participants used medical services in the past 1 month. In the past 6 months, 68.4 percent of participants had used medical services, and in the past 12 months, 73.8 percent of participants had used medical services.

### 3.3. Factors Associated with Older Adults' PHC Services Utilization

#### 3.3.1. In the Past 1-Month Period

Participants who used significantly more PHC services in the past month were those who (a) had no formal education or had a primary school education, (b) were nonsmokers, (c) had a chronic illness, (d) had perceptions of poorer health status, and (e) had symptoms or poor physical health (lower SF-12v2 PCS score) (see Table 2).

#### 3.3.2. In Past 6-Month Period

Factors significantly associated with increased use of PHC services were (a) increasing age, (b) being unemployed or retired, (c) having no formal education or only a primary school education, (d) being nonsmokers, (e) having a chronic illness, (f) those having poor self-rated general health status today, and (g) those having a poor self-rated general health status in the past 6 months (see Table 2).

#### 3.3.3. In Past 12-Month Period

Significantly greater use of PHC services was associated with (a) increasing age, (b) being unemployed or retired, (c) having no formal education or only a primary school education, (d) being nonsmokers, (e) having a chronic illness, (f) those having poor self-rated general health status today, and (g) those having poor self-rated general health status in the past 12 months (see Table 2).

### 3.4. Factor That Predicted Primary Health Care Service Utilization

The variable associated with PHC services utilization in the past 1-, 6-, and 12-month period was chronic illness (OR 13.324, 95% CI 3.614–49.128), (OR 19.634, CI 7.679–50.203), and (OR 17.915, 95% CI 6.974−0.023), respectively (see Table 3).

## 4. Discussion

### 4.1. Utilization Patterns

For older adults in three catchment areas of the Irbid governorate of Jordan, data on medical services utilization in the past 1, 6, and 12 months were not consistent. The findings showed that rate of medical services use by older adults in the past one month was less than 50%, which was consistent with a study in Hong Kong [8]. In the past 6 months, the findings showed that two-thirds of older adults used PHC services for medical health services, which differed from rates reported by Chou and Chi [7] study. In the past 12 months, the findings indicated that three-quarters of older adults used PHC services for medical health services, which is close to the Estonian study [9]. The high rate of medical service utilization in past 12 months compared with rate of utilization in the past 6-month and 1-month periods in the present study can be accounted for by the use of a longer time period. This would have increased the chances of including those who rarely used PHC services. Further studies need to be conducted on the utilization of more and different types of health care service in the changing health care context.

### 4.2. Factors Associated with PHC Service Utilization

#### 4.2.1. Predisposing Factors

In the present study, older age was not associated with PHC service utilization in the past 1 month, similar to that found in Spain [10]; however, older age was positively associated with PHC service utilization in the past 6 and 12 months. In contrast, older age in Thailand was associated with fewer health care services use in the past year [11]. Lower education level was associated with greater PHC service utilization in the past 1, 6, and 12 months, which was also found in other studies [10, 11]. Those who were unemployed or retired used more PHC services than those employed in the past 6 and 12 months. As limited PHC literature includes older adults, employment status was among the factors not included in many studies. However, similar results were observed in Ghana [13]. Nonsmoking older adults used more PHC services in the past 1, 6, and 12 months compared with smokers. This result was consistent with a previous study [10].

#### 4.2.2. Needs Factors

Chronic illness was significantly associated with PHC service utilization in the past 1, 6, and 12 months, consistent with studies in Thailand and Spain [10, 11]. Cognitive impairment was not associated with PHC service utilization in the past 1, 6, and 12 months which is in contrast to earlier findings [12, 13]. This disagreement may be attributed to the fact that only 3.2% of the sample had cognitive impairment in this study and due to different measures being used across studies. The perceived general health status factors were found to be negatively associated with PHC service utilization, indicating that participants who perceived that they were in poor health on the day of the interview tended to use more PHC services during the past 1, 6, and 12 months. One explanation may be that older adults, especially those who have chronic illnesses, tend to go to health centers mainly to obtain their prescribed medication. Also, both aging and many chronic illnesses are associated with disabilities and complications that are difficult to manage without utilizing health services. Also, chronicity does not portend improvement over a 1-year time period.

Further studies need to be conducted to confirm that perceived general health status today correlates with PHC service utilization of older adults. Also, those older adults who perceived that they were in poor health, in particular, poorer physical health (PCS score) in the past 1 month, tended to use more PHC services in the past 1 month. This result disagrees with previous studies [10, 13]. Older adults who perceived that they were in poor general health status in the past 6 and 12 months used more PHC services in those time periods and this has been found previously [10].

### 4.3. Predictors of PHC Services Use in the Past 1, 6, and 12 Months

Age was not a significant predictor of PHC services utilization in the past 1, 6, and 12 months, in contrast to a study in Ghana [13], and education level was not a significant predictor of PHC services utilization in the past 1, 6, and 12 months, which was similar to a South Korean study [12], and neither was employment status a significant predictor of PHC services utilization in the past 1, 6, and 12 months, consistent with the findings by [13]. Likewise, tobacco use was not a significant predictor of PHC services utilization in the past 1, 6, and 12 months, similar to a Spanish study [10].

Chronic illness was the strongest significant predictor of PHC services utilization in the past 1, 6, and 12 months. Multivariate analysis indicated that older adults who had chronic illnesses were 13.3, 19.6, and 17.9 times more likely to use PHC services than those who did not have chronic illnesses in the past 1, 6, and 12 months, respectively. This finding is consistent with another study [7]. The findings in this study can be explained by the fact that the majority of the older adults who used PHC services in the past 1, 6, and 12 months had at least one chronic illness, and chronic illnesses need continuous treatment. These data are important for planning purposes for the PHC service centers and suggest that patients with one or more chronic illnesses should be identified so that the PHC can be responsive to their continuous needs.

Perceived general health today was not a significant predictor of PHC services utilization in the past 1, 6, and 12 months. Likewise, the PCS score was not a significant predictor of PHC services utilization in the past 1 month. This finding diverges from a prior study [7]. One justification for this difference could be related to differences in measures that were used to assess the self-evaluation of physical health and to differences in recall periods in assessing the utilization.

### 4.4. Limitations

Health care services use, history of chronic illnesses, and perceived health status of participants were self-reported. However, several attempts were made to reduce the possibility of recall bias: (a) the study used different recall periods (1, 6, and 12 months), (b) PHC services use and history of chronic illnesses were measured as a dichotomous variable, which is easier to recall, and (c) the study participants were community dwelling older adults with a relatively low frequency of cognitive impairment (about 3.2% had evidence of cognitive impairment based on the ECAQ) [24]. Also, the results cannot be generalized to PHC services in other governorates or in the entire country.

## 5. Conclusion

About three-fourths of older adults in this study reported having at least one chronic illness. Hence, there was an unusually high rate of chronic illness among the participants in this study. In terms of utilization patterns, the findings reflected high utilization rates of PHC services among older adults over a one-year period. The present study identified several predisposing and need factors that were associated with PHC service utilization. However, the strongest predictor of PHC service utilization was a medical history of chronic illnesses.

The results of this study add to the body of knowledge in geriatric nursing regarding older adults' health seeking behavior which will help in developing an effective nursing care programs to promote well-being in Jordanian older adults. Good health care planning requires an understanding of PHC service use, and the approach used in this study could be replicated throughout Jordan. As such, it would enable the Ministry of Health in Jordan to gather relevant data necessary to provide appropriate health care services for older adults that are both efficient and cost-effective. Moreover, such data could help planning for healthier lifestyles throughout the life course and to build comprehensive and preventative health care programs in the PHC centers that will promote better health for older adults, instead of the current focus on cure and/or medication prescriptions.

## Figures and Tables

**Table 1:** Descriptive statistics of the predisposing, enabling, and need variables of older adult participants (*n* = 190).

Variables	%	Mean (S.D.)
*Predisposing variables *		
Age (in years)		64.6 (9.7)
Gender		
Male	57.4	
Female	42.6	
Marital status		
Married	88.4	
Widow	11.6	
Education level		
No education	36.8	
Primary school education	42.1	
Secondary and higher education	21.1	
Employment status		
Unemployed	55.3	
Retired	41.1	
Employed	3.7	
Tobacco user		
Nonsmoker	71.1	
Smoker	28.9	

*Enabling variables *		
Health insurance		
Uninsured	6.3	
Insured	93.7	
Income (Jordanian Dinars per month)		218.2 (88.7)

*Need variables *		
Chronic illnesses		
No chronic illnesses	27.9	
Have chronic illnesses	72.1	
Cognitive impairment		
No cognitive impairment	96.8	
Have cognitive impairment	3.2	
Perceived general health status today		6.61 (1.3)
Perceived general health status in the last 1 month		
PCS score		41.28 (11.0)
MCS score		50.46 (7.3)
Perceived general health status in the past 6 months		6.46 (1.3)
Perceived general health status in the past 12 months		6.64 (1.3)

**Table 2:** Factors associated with primary health care services utilization of older adults in the past 1, 6, and 12 months.

Variable	Utilization 1 month	Utilization 6 months	Utilization 12 months
*Predisposing variables *			
Age	0.134	0.229^**^	0.205^**^
Gender	−0.093	−0.105	−0.100
Marital status	0.129	0.069	0.017
Education level	−0.220^**^	−0.200^**^	−0.240^**^
Employment status	0.158	0.178^*^	0.218^**^
Tobacco use	−0.162^*^	−0.166^*^	−0.145^*^

*Enabling variables *			
Health insurance	0.087	0.056	0.002
Income	−0.135	−0.073	−0.111

*Need variables *			
Chronic illnesses	0.453^**^	0.663^**^	0.650^**^
Cognitive impairment	−0.030	0.058	0.034
Perceived general health status today	−0.272^**^	−0.355^**^	−0.373^**^
Perceived general health status in last 1 month:			
PCS score	−0.377^**^	—	—
MCS score	−0.106		
Perceived general health status in last 6 months	—	−0.409^**^	—
Perceived general health status in the last 12 months	—	—	−0.306^**^

^*^Correlation is significant at the 0.05 level (2-tailed).

^**^Correlation is significant at the 0.01 level (2-tailed).

**Table 3:** Binary logistic regression analysis of predictors of primary health care service utilization of older adults in the past 1, 6, and 12 months.

	PHC service utilization in the past 1 month			PHC service utilization in the past 6 months			PHC service utilization in the past 12 months		
	OR	CIs		OR	CIs		OR	CIs	
		Lower	Upper		Lower	Upper		Lower	Upper
*Predisposing factors *									
Age	0.98	0.94	1.02	1.00	0.95	1.05	0.97	0.92	1.03
Education level									
No education	1.37	0.37	5.03	0.76	0.14	4.00	3.26	0.56	18.80
Primary education	0.66	0.24	1.82	0.45	0.13	1.51	1.50	0.44	5.07
Secondary and higher education^#^	1.00	—	—	1.00	—	—	1.00	—	—
Employment status									
Unemployed	1.49	0.11	19.59	1.99	0.20	19.5	2.62	0.23	29.02
Retired	1.40	0.11	17.40	1.82	0.20	16.1	4.57	0.44	47.19
Employed^∧^	1.00	—	—	1.00	—	—	1.00	—	—
Tobacco use	0.78	0.34	1.77	0.78	0.30	2.03	0.83	0.30	2.30

*Need factors *									
Chronic illnesses	13.3^**^	3.61	49.12	19.6^**^	7.67	50.2	17.9^**^	6.97	0.02
Perceived general health today	0.92	0.67	1.26	1.03	0.61	1.75	0.65	0.42	1.03
PCS score	0.97	0.93	1.02						

Self-perceived general health in the past 6 months				0.66	0.37	1.17			
Self-perceived general health in the past 12 months							0.96	0.62	1.48

^#^Reference group.

^∧^Reference group.

*Note.* PCS: physical component summary score of SF-12v2.

OR: odds ratio.

CIs: 95% confidence interval.

^**^OR statistics is significant at the 0.001 level.
